# Cardiovascular health, traffic-related air pollution and noise: are associations mutually confounded? A systematic review

**DOI:** 10.1007/s00038-013-0489-7

**Published:** 2013-07-26

**Authors:** Louis-François Tétreault, Stéphane Perron, Audrey Smargiassi

**Affiliations:** 1 Département de santé environnementale et santé au travaill, University of Montreal, 2375 Chemin de la Côte-Sainte-Catherine, Montréal, QC H3T 1A8 Canada; 2 Département de médecine sociale et préventive, University of Montreal, 1301 Sherbrooke est, Montreal, QC H2L 1M3 Canada; 3 Direction de santé publique de l’Agence de la santé et des services sociaux de Montréal, Quebec, Canada

**Keywords:** Noise, Air pollution, Confounding, Cardiovascular

## Abstract

**Objectives:**

This review assessed the confounding effect of one traffic-related exposure (noise or air pollutants) on the association between the other exposure and cardiovascular outcomes.

**Methods:**

A systematic review was conducted with the databases Medline and Embase. The confounding effects in studies were assessed by using change in the estimate with a 10 % cutoff point. The influence on the change in the estimate of the quality of the studies, the exposure assessment methods and the correlation between road noise and air pollutions were also assessed.

**Results:**

Nine publications were identified. For most studies, the specified confounders produced changes in estimates <10 %. The correlation between noise and pollutants, the quality of the study and of the exposure assessment do not seem to influence the confounding effects.

**Conclusions:**

Results from this review suggest that confounding of cardiovascular effects by noise or air pollutants is low, though with further improvements in exposure assessment, the situation may change. More studies using pollution indicators specific to road traffic are needed to properly assess if noise and air pollution are subjected to confounding.

## Introduction

Studies have shown that exposure to road traffic noise and air pollutant emissions can lead to adverse health effects such as annoyance (World Health Organisation (2011), sleep disturbance (Pirrera et al. 2010), respiratory problems (Health Effects Institute Panel on the Health Effects of Traffic-Related Air Pollution 2010) and cancer (Beelen et al. 2008). A few studies (Finkelstein et al. 2004; Gan et al. 2010; Hoffmann et al. 2006, 2009; Maheswaran and Elliott 2003) have also reported associations with cardiovascular (CV) outcomes. CV health effects could be explained either by noise or air pollution associated with traffic.

On the one hand, there is evidence linking traffic noise to ischemic heart diseases (World Health Organisation 2011; Babisch and Kamp 2009; Babisch 2006) and hypertension (World Health Organisation Regional Office for Europe 2011; van Kempen and Babisch 2012). On the other hand, there is also evidence linking traffic-generated air pollution to CV diseases (Hoek et al. 2002; Brook et al. 2010). However, in a recent report of the Health Effects Institute Panel on the Health Effects of Traffic-Related Air Pollution 2010), the evidence of the effects of traffic-related air pollution on cardiovascular mortality was considered suggestive, but not sufficient. Nonetheless, there is increasing evidence connecting air pollution to overall cardiovascular mortality (Chen et al. 2013), myocardial infarction (Nuvolone et al. 2011; Rosenlund et al. 2006), atherosclerosis (Adar et al. 2013) and atrial fibrillation (Liao et al. 2010). Although noise or air pollution can confound the effect of each other, the underlying physiological mechanisms are likely different.

A few mechanisms are postulated to explain the effect of noise on CV diseases. Noise can act as a general stressor disturbing the body homeostasis through the “stress syndrome” (Babisch and Kamp 2009; Westman and Walters 1981; World Health Organisation Regional Office for Europe 2011; Amato et al. 2010; Gan et al. 2012). Noise can induce stress by two different pathways. The stress response generated by the direct pathway consists of a neural activation by the noise. In the indirect pathway, the activation of the stress response is created by a cognitive interpretation of the noise (Westman and Walters 1981; Gan et al. 2012). The activation of this pathway can be influenced by the perception of the noise, the perceived control over the sound and noise sensitivity of the subjects (European Environment Agency (EEA) 2010). There are also pathways by which noise could increase the risk of cardiovascular diseases. One of those is the disruption of sleeping patterns. Studies have associated the lack of reduction of blood pressure (BP) during the night (“BP dipping”) caused by noise with an increased risk of cardiovascular outcomes (Vardeny et al. 2011; Haralabidis et al. 2011). It is also suggested that short sleep durations may result in higher ghrelin and lower leptin concentrations (Taheri et al. 2004). The deregulation of those hormones linked with appetite regulation could potentially lead to obesity and higher risk of cardiovascular diseases.

For air pollution, several pathways are hypothesized to explain its impact on CV diseases. Firstly, air pollutants could generate an increase in lung oxidative stress and inflammation. Some pollutants could also migrate through the pulmonary epithelium into blood. Those pathways could lead to a systemic and vascular inflammation, increasing the risk of hypertension and thrombogenesis. Another hypothesized mechanism is the activation of the pulmonary reflex by particulate matter leading to the activation of the sympathetic system. A chronic activation of this system could lead to hypertension, plaque instability and cardiac arrhythmias (Brook et al. 2004; Burgan et al. 2010; Mills et al. 2007).

As motor vehicles are the predominant source of both air pollution and noise in many cities (Allen and Adar 2011), the reported associations between road traffic exposure and CV diseases could be influenced by a confounding or interaction between those two pollutants. Yet, few studies have aimed to untangle the possible effects of road traffic noise and air pollution. This is fundamental to better steer public health interventions and policies aimed at reducing CV effects of road traffic. For example, if road traffic induces CV effects through noise levels, then regulations to reduce car air pollutant emissions may not tackle the problem and zoning by-laws could be more appropriate. In this article, we reviewed epidemiological evidences that looked at the confounding effect of one traffic-related exposure (noise or air pollution) on the association between the exposure to traffic-related noise or air pollution and CV outcomes.

## Methods

### Data sources

The bibliographic databases used were Medline and Elsevier Embase on the Ovid SP portal. Only studies published until November 2012 were considered. No other temporal limitation was applied.

### Extraction strategy

The strategy used to conduct this review consisted of a combination of keywords representing three distinct categories: (1) exposure to traffic air pollution, (2) traffic noise exposure and (3) cardiovascular outcomes (see “Appendix” for specific keywords). Only peer review articles written in English or French on road traffic were reviewed. The studies were then selected manually according to the following exclusion criteria:

- Commentaries, editorials, review articles
- Studies not related to road traffic
- Studies not performed on humans
- Studies with no simultaneous exposure to noise and air pollutants
- Studies with no assessment of cardiovascular effects
- Studies not reporting the impact of confounding variables

Studies using the same cohort but assessing different CV outcomes were included. The references of each selected article were consulted to ensure that all pertinent information was gathered in our review. Experts in the field were also consulted to see if further articles could be added. Finally, gray literature (OAIster database, WHO and the New York Academy of Medicine) was also consulted. Studies presenting the associations before and after the authors controlled for the co-variables of interest (e.g., traffic noise or air pollution) were reviewed.

The articles selected were then separated into two categories: studies of CV outcomes where the noise exposure effect was adjusted for air pollutants and studies evaluating the effect of air pollutants, adjusted for the noise effect. In this review, cardiovascular findings of the original studies are reported before and after the control of either road traffic noise or air pollution effects. The effect of the supposed confounders was assessed using the percentage of variation in the estimate as follows: the confounding effect (C) of noise or air pollution was evaluated by a change in the point estimate (CIE) with a cutoff point of 10 % (Eq. 1) (Bliss et al. 2012; Vittinghoff et al. 2012).

1 $$ \frac{{ {\text{Adjusted}}\; {\text{point}}\; {\text{estimate}} - {\text{Unadjusted}}\; {\text{point}}\; {\text{estimate}}\; \times 100=  {\text{C}}}} {{\text{Unadjusted}}\; {\text{point}}\; {\text{estimate}}} $$

To assess the quality of the study and identify possible biases within the studies reviewed, two authors (LFT and SP) reviewed independently each study. The methodology applied has been described previously (Perron et al. 2012). Briefly, we considered the study’s design selection and classification bias relevant to our review. For each type of bias, the distinction between major and minor biases was assessed qualitatively by both authors. We defined major biases as those that could invalidate the results presented in the study. Minor biases are expected to affect the association studied, but unlikely to invalidate the conclusion.

## Results

**Fig. 1:**
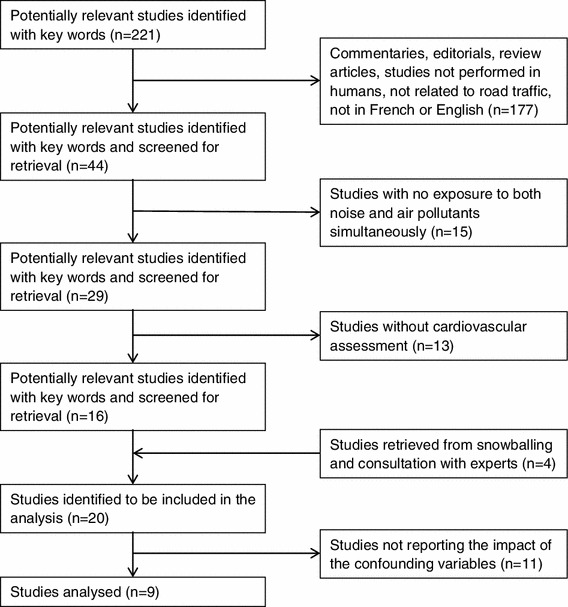
Stages of the selection of studies for analysis

Figure 1 presents a flowchart describing the stages of selection for the studies used in this review. Our keyword search yielded 221 starting articles ("Appendix"), which were reduced to 16 articles by applying the various inclusion and exclusions criteria. A large number of irrelevant articles had to be sifted by hand because of the double meaning of the terms sound and noise. We added four articles identified by snowballing and expert consultation. Of these 20 articles, a final 11 studies were discarded because it was impossible to assess the impact of the confounding variables of interest (Fig. 1). We did not find any document in the gray literature that could benefit this review.

Nine studies evaluating the relationship between CV outcomes, noise and traffic-related air pollution met our criterion (Beelen et al. 2009; Clark et al. 2012; de Kluizenaar et al. 2007; Dratva et al. 2012; Gan et al. 2012; Selander et al. 2009; Sorensen et al. 2011, 2012a, b). Of those articles, one assessed CV disease mortalities in general (Beelen et al. 2009), four assessed ischemic heart diseases (Beelen et al. 2009; Gan et al. 2012; Selander et al. 2009; Sorensen et al. 2012a), two assessed cerebrovascular diseases (Beelen et al. 2009; Sorensen et al. 2011), four looked at blood pressure or hypertension (Clark et al. 2012; Dratva et al. 2012; Sorensen et al. 2012b; de Kluizenaar et al. 2007), and one article was identified for both heart failure and cardiac dysrhythmia (Beelen et al. 2009).

Tables 1 and 2 present the associations between cardiovascular outcomes and either road traffic air pollution or noise. The tables also summarize the population characteristics of each study, the health outcomes monitored, as well as the noise and air pollutants indicators used. When available, the background exposure levels to pollution and noise of the subjects were reported.

**Table 1:** Estimation of the effects of exposure to noise levels on cardiovascular mortality and morbidity while controlling for air pollution (Sweden [1992–1994], Denmark [1993–2006], Canada [1994–2002], Switzerland [2002–2003], Netherlands [1997–1998] and, [1987–1996])

Studies		Characteristics of the study^a^	Traffic noise exposure (noise indicator) [validation of the model]	Air pollution exposure (air pollution indicator) [validation of the model]	Exposure levels	Health outcome (definition)	Main findings: (95 % confidence interval)	Percentage of change in the estimate
de Kluizenaar et al. (2007)	Groningen sample:	Cross-sectional *N*: 40,856 28–75 years Groningen, Netherlands 1997–1998	Dispersion model : The Standaart Kartering Method 2 implemented in Urbis (*L*_den_) [no information on validation]	Dispersion models: (i) Local traffic contribution from the model CAR II (ii) The Gaussian dispersion model “Pluim” (PM_10_) [no information on validation]	Average (SD) *L*_den_ in the Groningen sample: No AHT: 53.3 (6.9) dB(A) AHT: 54.6 (7.0) dB(A) Median (5–95 percentile) PM_10_ level in the Groningen sample: No AHT: 33.5 (32.8–37.5) μg/m^3^ AHT: 33.6 (32.9–37.6) μg/m^3^	Self-reported antihypertensive medication intake	OR per 10 dB(A) increase Full sample OR_A_ 1.01 (0.96–1.06) OR_P_ 1.03 (0.96–1.11) Subgroup 45–55 years OR_A_ 1.08 (0.97–1.20) OR_P_ 1.19 (1.02–1.40)	Full sample 1.98 % Subgroup 45–55 years 10.19 %
	PREVENT cohort sub sample	Cross-sectional *N*: 8,592 28–75 years Groningen, Netherlands 1997–1998				Hypertension: use of antihypertensive medication (pharmacy record) or systolic blood pressure ≥140 and diastolic blood pressure ≥90 (mean of the last 2 measurements from the 2 visits)	OR per 10 dB(A) increase Full sample OR_A_ 1.07 (0.98–1.18) OR_P_ 1.08 (0.95–1.23) Subgroup 45–55 years OR_A_ 1.27 (1.08–1.49) OR_P_ 1.39 (1.08–1.77)	Full sample 0.93 % Subgroup 45–55 years 9.45 %
Beelen et al. (2009)		Cohort *N*: 117,528 55–69 years Netherlands 1987–1996	Dispersion model :Empara (*L*_den_) [Measured vs. Estimated: on average <2–3 dB(A)]	Land use regression (black smoke) [*R* ^2^ = 0.59]	Average *L*_den_ level (SD) : 52 (7) dB(A) Black smoke average level: 13.9 (2.2) μg/m³ NO_2_ average level: 30 mg/m^3^	Mortality from: ischemic heart disease, cardiovascular disease, cerebrovascular disease, heart failure and cardiac dysrhythmia (ICD 9 for 1986–1995 and ICD 10 for 1996)	RR compared to a reference category of ≤50 dB(A) Overall cardiovascular mortality RR_A_ 1.25 (1.01–1.53) RR_P_ 1.17 (0.94–1.45) Ischemic heart disease mortality RR_A_ 1.15 (0.86–1.53) RR_P_ 1.01 (0.74–1.36) Cerebrovascular mortality RR_A_ 0.88 (0.52–1.50) RR_P_ 0.95 (0.55–1.66) Heart failure mortality RR_A_ 1.99 (1.05–3.79) RR_P_ 1.90 (0.96–3.78) Cardiac dysrhythmia mortality RR_A_ 1.23 (0.50–3.01) RR_P_ 1.23 (0.48–3.13)	Overall cardiovascular mortality 6.40 % Ischemic heart disease mortality 12.17 % Cerebrovascular mortality 7.95 % Heart failure mortality 4.52 % Cardiac dysrhythmia mortality 0.00 %
Selander et al. (2009)		Case–control *N*: 2,095 (controls) + 1,571 (cases) 45–70 years Stockholm country, Sweden 1992–1994	Dispersion model: simplified Nordic prediction method (*L*_A,eq,24h_) [No information on validation]	Dispersion model: Gaussian Air Quality Dispersion model (NO_2_) [No information on validation]	*L*_A,eq,24h_: NA NO_2_: median level 12.9 μg/m^3^ for controls Median level for cases: NA	Myocardial infarction (coronary records, hospital discharge register and the National Cause of Death at statistic Sweden)	OR compared to a reference category of ≤50 dB Full sample OR_P_ 1.12 (0.95–1.33)	7 %
Sorensen et al. (2011)		Cohort *N*: 51,485 55–64 years Copenhagen or Aarthus, Denmark 1993–2006	Dispersion model: Sound plan with the Nordic prediction method, DANSIM and INM 3 (*L*_den_) [Measured vs. Estimated: on average 0.2 dB]	Dispersion model: Danish AirGis (NO_X_) [*R* ^2^ = 0.75]	Median *L*_den_ (5–95 percentile) : <64.5 years 57.8 (NA) dB(A) and ≥64.5 years 58.2 (NA) dB(A) NO_X_ 5–95 percentile) median levels at *L*_den_ ≤60 dB 18.5 (14.1–28.3) μg/m^3^ and *L*_den_ >60 dB 34.3 (16.9–137) μg/m^3^	Stroke (hospital discharge register ICD 8 and 10)	IRR per 10 dB(A) increase Full sample IRR_A_ 1.10 (1.03–1.18) IRR_P_ 1.14 (1.03–1.25)	3.64 %
Sorensen et al. (2012a)		Cohort *N*: 50,614 55–64 years Copenhagen or Aarthus, Denmark 1993–2006	Dispersion model: Sound plan with the Nordic prediction method, DANSIM and INM 3 (*L*_den_) [Measured vs. Estimated: on average 0.2 dB]	Dispersion model: Danish AirGis (NO_X_) [*R* ^2^ = 0.75]	Median *L*_den_ (5–95 percentile) 56.4 (48.5–70.1) dB(A) Median NO_X_ (5–95 percentile) 20.8 (14.4–88.0) μg/m^3^	Myocardial infarction (ICD 10)	IRR per 10 dB(A) increase Full sample (per 10 dB[A]) IRR_crude_ 1.10 (1.03–1.19) IRR_Adj_ 1.12 (1.02–1.22)	1.82 %
Gan et al. (2012)		Cohort *N*: 445,868 45–85 years Vancouver Canada 1994–2002	Dispersion model: CadnaA base model using the EMME/2 for traffic volume (*L*_den_) [No information on validation]	Land use regression (NO_2_, PM_2.5_ and black carbon) [NO_2_: *R* ^2^ = 0.56 PM_2.5_ *R* ^2^ = 0.52 Black carbon NA]	Average *L*_den_ (SD): 63.4 (5.0) dB(A) PM_2.5_ average level (SD): 4.10 (1.64) μg/m^3^ NO_2_ average level (SD): 32.3 (8.1) μg/m^3^ NO_X_ average level (SD): 32.2 (12.0) μg/m^3^ Black carbon average level (SD): 1.50 (1.1) 10^−5^/m	Ischemic heart diseases mortalities (ICD-9 and ICD-10)	RR per increase of 10 dB(A) PM_2.5_ RR_A_ 1.13 (1.06–1.21) RR _P_ 1.13 (1.06–1.21) NO_2_ + PM_2.5_ RR_A_ 1.13 (1.06–1.21) RR _P_ 1.12 (1.05–1.21) Black carbon + NO_2_ + PM_2.5_ RR_A_ 1.13 (1.06–1.21) RR _P_ 1.09 (1.01–1.18)	PM_2.5_ 0.00 % NO_2_ + PM_2.5_ 0.88 % Black carbon + NO_2_ + PM_2.5_ 3.54 %
Dratva et al. (2012)		Cross-sectional *N*: 6,450 28–72 years Switzerland 2002–2003	Dispersion model: SONABASE (*L*_Day_ and *L*_night_) [Measured vs. Estimated: on average ± 2.6 dB(A) (day) ± 3.1 dB(A) (night)]	Dispersion model: PolluMap Gaussian dispersion model (NO_2_) [*R* ^2^ = 0.72]	Average (SD) *L*_day_ : 50.5 (7.2) dB(A) Average (SD) *L*_night_ : 38.7 (7.8) dB(A) Average (SD) levels for: NO_2_: 23.0 (9.9) μg/m^3^ PM_10_: 21.3 (7.1) μg/m^3^	Blood pressure (measured by the Riva-Rocci method by trained field workers)	Increase in BP per 10 dB (A) Night time systolic BP * β*_A_: −0.01 (−0.6 to 0.59) * β*_AP_: 0.15 (−0.48 to 0.77) Nighttime diastolic BP * β*_A_: −0.05 (−0.41 to 0.30) * β*_AP_: −0.15 (−0.36 to 0.39) Daytime systolic BP * β*_A_: −0.11 (−0.68 to 0.47) * β*_AP_: 0.05 (−0.56 to 0.07) Daytime diastolic BP * β*_A_: −0.10 (−0.44 to 0.24) * β*_AP_: −0.04 (−0.40 to 0.33)	Nighttime systolic BP 1,600 % Nighttime diastolic BP 200 % Daytime systolic BP 145.45 % Daytime diastolic BP 60 %

^a^ Final sample sizes used for analysis

*OR* odds ratio, *NA* not available, *IRR* incident rate ratio, *RR* risk ratio, *A* adjusted for some of the following potential confounding factors: age, education, employment, marital status, study area, mean pulse, hearing impairment, noise at work, crowding, home ownership, mother’s educational level, language spoken at home, parental support for schoolwork, classroom window glazing, body mass index, cuff size, room temperature, birth weight, parental high blood pressure, prematurity, smoking status, family history of CVD, physical inactivity smoking intensity, intake of fruits, intake of vegetables, intake of coffee, alcohol intake diabetes, antihypertensive medication, high blood pressure, long-standing illness and other comorbidity (see original article for details), *P* adjusted for some potential confounding factors and air pollution levels, *AHT* antihypertensive treatment, *SD* standard deviation, *β* regression coefficient, *PM*_*2.5*_ particles with a diameter of 2.5 μm or smaller, *PM*_*10*_ particles with a diameter of larger than 2.5 μm, but smaller than 10 μm, *NO*_*2*_ Nitrogen dioxide, *NO*_*X*_ Nitrogen oxide, *dB(A)* A-weighted decibels, *L*_day_ integrated A-weighted sound level over 16 h (0600–2200), *L*_night_ integrated A-weighted sound level over 8 h (2200–0600), *L*_A,eq,24h_ integrated A-weighted sound level over 24 h, *L*_den_ integrated A-weighted sound level over 24 h (day, evening and night) in which sound levels during the evening (1900–2300 hours) are increased by 5 dB(A) and those during the night (2300–0700 hours) by 10 dB(A)

**Table 2:** Estimation of effects of exposure to ambient air pollutants on cardiovascular mortality and morbidity while controlling for noise (Netherlands [1987–1996], Denmark [2000–2002] and UK [2001–2003])

Studies	Characteristics of the study^a^	Traffic noise exposure (noise indicator) [validation of the model]	Air pollution exposure (air pollution indicator) [validation of the model]	Exposure levels	Health outcome (definition)	Main findings : air pollutions effects adjusted for noise effects	Percentage of change in the estimate
Beelen et al. (2009)	Cohort *N* 117,528 55–69 years Netherlands 1987–1996	Dispersion model :Empara (*L*_den_) [Measured vs. Estimated: on average <2–3 dB(A)]	Land use regression (black smoke) [*R* ^2^ = 0.59]	Average *L*_den_ level (SD) : 52 (7) dB(A) Black smoke average level: 13.9 (2.2) μg/m³ NO_2_ average level : 30 mg/m^3^	Mortality from: ischemic heart disease, cardiovascular disease, cerebrovascular disease, heart failure and cardiac dysrhythmia (ICD 9 for 1986–1995 and ICD 10 for 1996)	RR for an increase of 10 μg/m^3^ of black smoke and adjusted for traffic intensity Overall cardiovascular mortality RR_A_ 1.11 (0.96–1.28) RR_PT_ 1.11 (0.95–1.28) [RR_A_ 1.01 (1.00–1.02) RR_PT_ 1.01 (0.99–1.02)]^†^ Ischemic heart disease mortality RR_A_ 1.01 (0.83–1.22) RR_PT_ 1.01 (0.83–1.22) [RR_A_ 1.00 (0.98–1.02) RR_PT_ 1.00 (0.98–1.02)]^†^ Cerebrovascular mortality RR_A_ 1.39 (0.99–1.94) RR_PT_ 1.41 (1.01–1.97) [RR_A _1.03 (1.00–1.07) RR_PT_ 1.03 (1.00–1.07)]^†^ Heart failure mortality RR_A_ 1.75 (1.00–3.05) RR_PT_ 1.76 (1.01–3.08) [RR_A_ 1.06 (1.00–1.12) RR_PT_ 1.06 (1.00–1.12)]^†^ Cardiac dysrhythmia mortality RR_A_ 0.96 (0.51–1.79) RR_PT_ 0.94 (0.50–1.76) [RR_A_ 1.00 (0.99–1.06) RR_PT_ 0.99 (0.93–1.06)]^†^	Overall cardiovascular mortality 0.00 % [0.00 %]^†^ Ischemic heart disease mortality 0.00 % [0.00 %]^†^ Cerebrovascular mortality 1.44 % [0.14 %]^†^ Heart failure mortality 0.57 % [0.06 %]^†^ Cardiac dysrhythmia mortality 2.08 % [0.21 %]^†^
Sorensen et al. (2012b)	Cohort (cross-sectional to assess the BP results) *N*: 44,436 55–64 years Copenhagen or Aarthus, Denmark 2000–2002	Dispersion model: Sound plan with the Nordic prediction method, DANSIM and INM 3 (*L*_den_) [Measured vs. Estimated: on average 0.2 dB]	Dispersion model: Danish AirGis (NO_X_) [*R* ^2^ = 0.75]	Median baseline *L*_den_ 5–95 percentile) 56.3 (48.4–70.0) dB(A) Median baseline NO_X_ (5–95 percentile) 20.2 (14.3–86.8) μg/m^3^	Difference in BP (mmHg)	Regression coefficient for a doubling in NO_X_ level Systolic BP * β*_A_: −0.39 (−0.64; −0.13) * β*_P_: −0.53 (−0.88; −0.19)	Systolic BP 35.90 %
Clark et al. (2012)	Cross-sectional *N*: 719 9–10 years UK 2001–2003	Dispersion model: simplified form of the UK standard calculation of road traffic noise (*L*_Day_) [No information on validation]	Dispersion model: King’s College London Emissions Toolkit (NO_2_) [Measured vs. Estimated: on average 2.4 ppb]	Average *L*_day_ : 50 dB(A) NO_2_ average level 42.73 μg/m^3^	Blood pressure measured using automatic blood pressure meters (OMORON 711)	Regression coefficient for an increase of 1 μg/m^3^ NO_2_ increase Systolic BP * β*_A_: 0.058 (−0.092 to 0.210) * β*_P_: 0.070 (−0.120 to 0.259) Diastolic BP * β*_A_: 0.033 (−0.084 to 0.151) * β*_P_: 0.088 (−0.059 to 0.236)	Systolic BP 20.69 % Diastolic BP 166.67 %

*RR* Risk ratio, *SD* standard deviation, *β* regression coefficient, *NO*_*2*_ Nitrogen dioxide, *NO*_*X*_ Nitrogen oxide, *dB(A)* A-weighted decibels, *L*_day_ Integrated A-weighted sound level over 16 h (0600–2200), *L*_den_ Integrated A-weighted sound level over 24 h (day, evening and night) in which sound levels during the evening (1900–2300 hours) are increased by 5 dB(A) and those during the night (2300–0700 hours) by 10 dB(A), *A* adjusted for some of the following potential confounding factors: age, education, employment, marital status, study area, mean pulse, hearing impairment, noise at work, crowding, home ownership, mother’s educational level, language spoken at home, parental support for schoolwork, classroom window glazing, body mass index, cuff size, room temperature, birth weight, parental high blood pressure, prematurity, smoking status, family history of CVD, physical inactivity smoking intensity, intake of fruits, intake of vegetables, intake of coffee, alcohol intake diabetes, antihypertensive medication, high blood pressure, long-standing illness and other comorbidity, *P* adjusted for potential confounding factors including air pollution levels

^a^ Final sample sizes used for analysis

^†^ Results for an increase of 1 µg/m^3^

Six studies used day–evening–night noise levels (*L*_den_), as indicators of exposure to noise levels from road traffic (Beelen et al. 2009; de Kluizenaar et al. 2007; Gan et al. 2012; Selander et al. 2009; Sorensen et al. 2011, 2012a, b). Others studies used equivalent noise levels over 24 h (*L*_eq,24h_) (Selander et al. 2009), night noise levels (*L*_night_) (Dratva et al. 2012) or daily noise levels (*L*_day_) (Clark et al. 2012; Dratva et al. 2012). All studies reported used dispersion noise models, but only four reported validation information on the model used (Beelen et al. 2009; Sorensen et al. 2011, 2012a, b). In those studies, the average difference between the estimated noise levels and measured noise levels was 3.1 dB or less. Regarding exposure to air pollutants, seven studies used nitrogen oxide (NO_x_ or NO_2_) as an indicator (Clark et al. 2012; Dratva et al. 2012; Gan et al. 2012; Selander et al. 2009; Sorensen et al. 2011, 2012a, b). Two articles reported results based on particulate matter (PM_10_ or PM_2.5_) (de Kluizenaar et al. 2007; Gan et al. 2012). Two studies used measurements of black smoke or black carbon to account for road traffic pollutants (Beelen et al. 2009; Gan et al. 2012). Of those studies, two applied land use regression models (Beelen et al. 2009; Gan et al. 2012) to assess the air pollution exposure, while the other studies employed dispersion models (Clark et al. 2012; de Kluizenaar et al. 2007; Dratva et al. 2012; Selander et al. 2009; Sorensen et al. 2011, 2012a, b). As noted in Tables 1 and 2, seven out of the nine studies reviewed reported validation information on their air pollution model (Beelen et al. 2009; Clark et al. 2012; Dratva et al. 2012; Gan et al. 2012; Sorensen et al. 2011, 2012a, b). The Clark et al. study reported an average difference between the estimated and the measured pollutant levels of 2.4 ppb. The remaining studies presented *R* ^2^ that ranged from 0.52 to 0.75. All articles presented average yearly outdoor exposure for both noise and air pollutant levels as a proxy of individual exposure. The correlations between traffic noise and air pollutants in the studies reviewed range from 0.16 to 0.72. It is surprising that studies where both noise and air pollutants were modeled, relying on some of the same variables (e.g., distance to traffic source), could generate low and disparate correlations.

### Quality of studies

Table 3 presents the assessment of the quality of the studies by LFT and SP. All studies, with the exception of Selander et al. (2009) and Dratva et al. (2012), only considered the exposure at home or at work/school, which we considered as a minor classification bias likely to be non-differential. Two studies also used medical administrative databases to identify the cause of death, potentially leading to another minor classification bias. Four studies reported response rates between 30 and 60 % which could lead to a selection bias and one did not report the response rate. Finally, four studies used cross-sectional designs to assess associations with cardiovascular outcomes reported in this review. Most studies contained less than four minor biases. We did not identify any major bias in the studies assessed.

**Table 3:** Quality assessment of the studies reviewed (UK [2001–2003], Sweden [1992–1994], Denmark [1993–2006], Canada [1994–2002], Switzerland [2002–2003], Netherlands [1997–1998] and, [1987–1996])

Author(s)	Selection biases		Classification biases		Study design
	Major	Minor	Major	Minor	
Beelen et al. (2009)	None	Approximately, 85 % of the population at baseline had no paid job.	None	Input data from 2000 for the noise model paired with the 1986 home address Cause of death based on non-validated medico administrative databases Exposure assessed with the residential address only	Case cohort
Sorensen et al. (2011)	None	Response rate between 30 and 60 %	None	Exposure assessed with the residential address only	Cohort
de Kluizenaar et al. (2007)	None	Response rate between 30 and 60 %	None	The air pollution indicator was not specific to road traffic Exposure assessed with the residential address only	Cross-sectional
Sorensen et al. (2012a)	None	Response rate between 30 and 60 %	None	Exposure assessed with the residential address only	Cohort
Sorensen et al. (2012b)	None	Response rate between 30 and 60 %	None	Exposure assessed with the residential address only	Cross-sectional^a^
Gan et al. (2012)	None	None	None	Cause of death based on non-validated medico administrative databases Exposure assessed with the residential address only	Cohort
Selander et al. (2009)	None	None	None	None	Case control
Dratva et al. (2012)	None	No direct information on response rate	None	None	Cross-sectional^a^
Clark et al. (2012)	None	Exclusion of 7 of the 9 school because of missing air pollution exposure	None	Exposure assessed at school only	Cross-sectional^a^

^a^ Design used for the CV outcome of interest

### Noise effects controlled for air pollution effects

As illustrated in Table 1, four studies assessed the association between noise levels and ischemic heart diseases while controlling for air pollutants. Gan et al. (2012) reported an association between death from coronary heart disease and a 10 dB(A) elevation of the *L*_den_ level of 1.13 (95 % CI 1.06, 1.21) before and 1.09 (95 % CI 1.01, 1.18) after controlling for NO_2_, PM_2.5_ and black carbon. Models adjusted for PM_2.5_ and NO_2_ only in Gan et al. (2012), produced small modifications of the point estimate (Gan et al. 2012). The IRR estimated by Sorensen et al. (2012a) for the association between the incidence of myocardial infarction and *L*_den_ was 1.10 (95 % CI 1.03, 1.19) per increase of 10 dB(A); this IRR increased to 1.12 (95 % CI 1.02, 1.22) after adjustment for NO_x_. Selander et al. (2009) only reported an association between myocardial infarction and *L*_eq,24h_ after controlling for NO_2_ (1.12 (95 % CI 0.95, 1.33) with the reference category <50 dB[A]). While Selander et al. (2009) did not present point estimates before and after adjusting for noise, the authors reported a 7 % change of the crude coefficient when compared with the model adjusted for air pollution. The percentage changes in the point estimates in the studies mentioned above were all below 10 % (ranging between 0 and 7 %). Beelen et al. (2009) also reported a reduction of the RR between ischemic heart disease mortality and annual *L*_den_ level after adjusting for black smoke and traffic intensity (from 1.15 (95 % CI 0.86, 1.53) to 1.01 (95 % CI 0.74, 1.36) with the reference category <50 dB[A]). However, this study was the only one that presented a variation of >10 % in the point estimate (12.17 %) following adjustment for air pollution.

For cerebrovascular diseases, Sorensen et al. (2011) published a positive association between hospitalization for stroke and *L*_den_ before (1.18 (95 % CI 1.11, 1.26) per 10 dB[A]) and after (1.14 (95 % CI 1.03, 1.25) per 10 dB[A]) controlling for NO_x_. The crude association between *L*_den_ level and cerebrovascular mortalities found in Beelen et al. (2009) was 0.88 (95 % CI 0.52, 1.50) and moved toward unity 0.95 (95 % CI 0.55, 1.66) after controlling for black smoke. Both studies reported a percentage change in their estimate of <10 % (respectively 3.64 and 7.95 %).

Two studies assessed the effect of noise on blood pressure. In the first (de Kluizenaar et al. 2007), reported associations between *L*_den_ and self-reported antihypertensive medication intake or hypertension were, respectively, 1.01 (95 % CI 0.96, 1.06) and 1.07 (0.98; 1.18) before controlling for PM_10_. Controlling for air pollutants for both outcomes resulted in a small change in the odds ratios, respectively, of 1.03 (95 % CI 0.96, 1.11) and 1.08 (95 % CI 0.95, 1.23). So adjusting for PM_10_ produced a CIE of 0.93 % for hypertension and 1.96 % for self-reported antihypertensive medication intake. Once stratified by age in both samples (the Groningen sample and the prevent cohort subsample), the only subgroup presenting significant associations was composed of individuals between 45 and 55 years old. In this age group, the percentage CIE was near our cutoff point for confounding effects (9.45 % in the prevent cohort and 10.19 % in the Groningen sample). The second study assessing blood pressure (Dratva et al. 2012) presented no significant association between road traffic noise (*L*_night_ and *L*_day_) and either systolic or diastolic blood pressure before and after adjustment for NO_2_. The regression coefficient did, however, vary extensively before and after adjustment for air pollutants resulting in CIE ranging from 60 to 1,600 %. The effects of noise on overall cardiovascular diseases, heart failure and cardiac dysrhythmia were reported only in Beelen et al. (2009). All associations in the final model were reduced or were identical after an adjustment for black smoke. The percentage CIE was <10 %.

The correlation between road traffic noise and air pollution reported in the studies on noise effects described above does not seem to influence the CIE produced by adjusting for air pollution levels. Studies that presented weak and high correlations (see “Appendix”) were both subject to large CIE. The quality of the approach used to estimate the confounder exposure levels (i.e., air pollution) does not appear to impact the CIE either. As presented in Table 1, the largest CIE were observed neither in studies with the small *R* ^2^ nor in those with the large *R* ^2^. Though CIE does not seem to be linked to the quality of the study (quantity of biases), cohort studies appear to generally report smaller CIE than studies using case–control or cross-sectional designs.

### Air pollution controlled for noise

As shown in Table 2, three studies assessed the association between air pollutant levels and cardiovascular diseases while controlling for noise levels. Beelen et al. (2009) reported associations between black smoke and mortality from overall cardiovascular diseases, heart failure ischemic diseases, cardiac dysrhythmia and cerebrovascular diseases. The percentage changes in the estimates for the associations reported in Beelen et al. (2009) ranged from 0.00 to 2.08 %, well below our predefined cutoff point for confounding effects. The two remaining studies reported associations with blood pressure. Sorensen et al. (2012b) presented a negative association between NO_x_ levels and systolic BP (−0.39 (95 % CI −0.64, −0.13) for doubling the 1 year concentrations). This association was stronger after adjustment for *L*_den_ (−0.53 (95 % CI −0.88, −0.19) (CI obtained from a personal communication with Mette Sorensen 01-06-2012). Adjusting for noise levels led to a 35.90 % change in the regression coefficient. In Clark et al. (2012), the regression coefficient representing the association between diastolic BP and NO_2_ varied from 0.033 (95 % CI −0.084, 0.151) to 0,088 (95 % CI −0.059, 0.236) per one point increase of NO_2_ (μg/m^3^); before and after adjusting for noise levels (*L*_day_). The regression coefficient for the association between systolic BP and NO_2_ increased from 0.058 (95 % CI −0.092, 0.210) to 0.070 (95 % CI −0.120, 0.259) per one point increase of NO_2_ (μg/m^3^), before and after adjusting for noise levels (*L*_day_). The modification of the regression coefficient for diastolic and systolic BP was, respectively, 166.67 and 20.69 %.

The correlation between road traffic noise and air pollution reported in the studies on traffic-related pollutants described above does not seem to influence the CIE produced by controlling for air pollution levels. The CIE also appears to be independent of the number of biases. On the other hand, studies with cross-sectional design presented higher CIE than the case–control study. Since none of the studies had validation information on noise exposure estimates, we could not assess the impact of the quality of the approach used to estimate the confounder exposure levels (i.e., noise) in these studies.

### Interaction

Regarding studies that assessed the interaction between air pollutant levels and noise on cardiovascular outcomes, only two were identified. Selander et al. (2009) did not report a significant interaction between annual NO_2_ levels and *L*_eq,24h_. Gan et al. (2012) did not find a statistically significant interaction between black carbon and noise levels (*L*_den_) for ischemic heart diseases.

## Discussion

This review aimed to assess the confounding effects of one traffic-related exposure (either noise or air pollutants) on the association between its counterpart and cardiovascular outcomes. In general, the results of the nine studies reviewed here showed that when associations between noise and CV diseases were adjusted for air pollutants, modifications of the point estimates for cardiovascular diseases were <10 %, after controlling for the air pollutants, with the exception of the studies by de Kluizenaar et al. (2007), Beelen et al. (2009) and Dratva et al. (2012) where the CIE was higher than our cutoff point for confounding effects. The Beelen et al. (2009) study reported a marked decrease of the strength of the association after adjustment for air pollution and traffic intensity. Yet, the simultaneous adjustment for traffic intensity and black smoke makes the evaluation of confounding by black smoke difficult in this study. Nonetheless, no association between road traffic and CV outcomes (before and after adjustment) was found in both the Dratva et al. (2012) and the Beelen et al. (2009) studies, rendering the CIE meaningless. By its definition, a confounder must modify the association between the exposure and the outcome. To confound, such associations must be present at least before or after adjustment. de Kluizenaar et al. (2007) reported CIE of <2 % in both the Groningen sample and the prevent cohort. However, CIEs nearing 10 % were observed in the 45–55 years subgroup, which could indicate the presence of a small confounding between the two exposures in this particular subgroup. Similar findings were found for associations between air pollutant levels and CV diseases, although the number of studies was limited (*N* = 3): controlling for noise levels either changed the point estimates for CV diseases by <10 % or the study did not present an association between the exposure and the outcome (before and after adjustment). Only the Sorensen et al. (2012b) study presented an indication of confounding by traffic noise in the association between NO_x_ levels and blood pressure. Nonetheless, overall these findings suggest an independent effect of noise and air pollution on CV diseases, particularly ischemic disease for which there were a greater number of studies. The review also points to the absence of comparability between studies. Most studies were difficult to compare because different noise or air pollution indicators were used, the pollution levels were assessed using different techniques and very few studies assessed comparable health outcomes.

In this review, we also tried to verify if the impacts of noise and air pollutants on CV were subject to the same interactive effects. Though both studies (Gan et al. 2012; Selander et al. 2009) that assessed interaction effects did not find any effect, one cannot conclude that there was an absence of interaction effect between noise and air pollutant levels with so few studies. This is particularly true in the light of the point raised by Selander et al. (2009) that the interaction analysis might have lacked power. It should also be noted that both studies used different noise indicators, the air pollutant or the cardiovascular outcome, to identify possible interaction effects. Those results do suggest, though, that if a multiplicative interaction exists, it is likely to be small.

Due to limitations of the literature, we cannot conclusively ascertain the independence of the effects of the two risks on any CV health outcome. Nonetheless, the results reported tend to indicate that the impacts of traffic noise and air pollution on cardiovascular outcome are distinct, or at least that they are not completely dependent on one another. Furthermore, the correlation between noise and air pollutant levels does not seem to influence the confounding effects. A wide range of correlations between noise and air pollutants were reported in the studies reviewed and this could be partially explained by differences in the urban structure at each location (building height, distance of buildings to sidewalks, street width, traffic intensity and distance to major road). This would suggest that confounding between traffic-related noise and air pollution is a study-dependant issue. However, high correlations between noise and pollutant levels were not associated with greater confounding effects in the studies reviewed. Additionally, the quality of the exposure assessment of the confounding variables and the quality of the studies (number of biases) do not seem to influence the confounding effects. However, the reported CIE seemed higher in studies that used a cross-sectional design. This may be because these studies mainly used linear regressions and presented regression coefficients. In fact, the approach that we used to assess confounding effects was developed for risk ratios and may not be applicable for linear regression. Nonetheless, this approach has been suggested by some authors in linear regression text books (Vittinghoff et al. 2012).

This review is subjected to a few limitations. First, only a small number of studies were available to be reviewed, which reduced the strength of our findings. Secondly, with any systematic review, the possibility of publication bias is present. We tried to minimize this bias by searching the gray literature and reviewing some non-English publications, but this bias cannot be excluded. We also tried to minimize the selective reporting bias, which could be a major one in this review. Indeed, the principal objective of most of the studies reviewed was not to assess the confounding effect between noise and air pollutants from road traffic. As shown in Fig. 1, more than half of the studies presenting the basic characteristics to be included did not report the impact of the co-variables of interest or the regression coefficients. It is also possible that some authors assessing the effect of either noise or air pollutant levels on CV diseases found that the corresponding co-variable was not significant and did not report it. This omission would, however, strengthen our results, as it would seem to indicate the independence of the effects of both exposures. Finally, the strategy used to assess the confounding effect in studies could also be criticized. The 10 % variation in the estimate is an arbitrary cutoff point that does not necessarily rule out confounding or inform on the statistical variability. Nonetheless, this cutoff point is widely used in the literature and was identified as the least biased in a simulation presented in Maldonado and Greenland (1993), in the absence of prior knowledge of confounders. Another potential limitation in our assessment of confounding is that the 10 % cutoff implies that effects are linear. In the case of nonlinear effect estimates, the absence of CIE does not preclude confounding (Janes et al. 2010). This might be the case if noise had a threshold effect, and future studies should address this.

To assess clearly the presence of confounding, future studies should use coherent noise and air pollution indicators. Those indicators should be chosen according to the effects examined. It would be better to use maximum noise levels (*L*_max_) or equivalent noise over 1 h (*L*_eq,1h_) to assess acute effects, and *L*_eq,24h_ to assess chronic effects. The use of *L*_den_ or *L*_DN_ as the noise metrics in the studies included in this review could be contested. Those noise metrics created to assess annoyance increase the weight noise levels occurring in evening or at night and are therefore not representative of the actual sound exposure. The source of the exposure is also an important factor to take into account while choosing an indicator. For a surrogate of all pollutants emitted by on-road traffic, one should use individual pollutants such as black carbon, NO_X_ and ultrafine particulate that are more source specific. More studies on the relationship between noise outdoor exposure levels and personal exposure levels should be conducted. This is important given that this relationship could differ between noise and air pollution. Furthermore, future studies should present adjusted results for both noise and air pollution modeled separately, so that the impact of the pollutant that was controlled for can be assessed. As shown in Tables 1 and 2, only few studies provided validation information for the exposure models used in their study. The absence of such information prevents the reader from judging the quality of the exposure assessment and thereby precluding the reader to judge from assessing the quality of the adjustments made. Finally, more studies are needed to find out if the confounding effect is specific to subcategories of CV outcomes. Ideally, those studies would need an epidemiologic design enabling them to assess the chronic effects of both traffic-related pollutants. We also recommend that those studies focus their research on ischemic heart diseases, hypertension or the fluctuation in blood pressure, for which mechanisms should yield more conclusive results.

### Conclusion

Results from this review suggest that confounding of cardiovascular effects by noise or air pollutants is low, on average, though heterogeneity across studies and areas within studies has been reported. The quality of exposure assessment of the confounding variable, the quality of studies as well as the correlation between noise and pollutant levels do not seem to influence the confounding effect, though with further improvements in exposure assessment, the situation may change. More studies using air pollution indicators specific to road traffic are needed to properly assess if road noise and pollutant effects on CV outcomes are subjected to the confounding effect of one another.

## Appendix

Search strategy

1. cardiovascular.mp. (906078)
2. hypertension.mp. (887805)
3. arterial tension.mp. (560)
4. blood pressure.mp. (753655)
5. arrythmia.mp. (783)
6. myocardial infraction.mp. (811)
7. stroke.mp. (382573)
8. vasoconstriction.mp. (79992)
9. ischemia.mp. (461795)
10. heart.mp. (2590525)
11. coagulation.mp. (227773)
12. arteries.mp. (364558)
13. blood flow.mp. (467842)
14. electrocardiogram.mp. (112029)
15. cardio^a^.mp. (1557392)
16. 1 or 2 or 3 or 4 or 5 or 6 or 7 or 8 or 9 or 10 or 11 or 12 or 13 or 14 or 15 (5132805)
17. noise.mp. (184815)
18. sound.mp. (142837)
19. 17 or 18 (309938)
20. 16 and 19 (31528)
21. air pollution.mp. (104657)
22. particles.mp. (301041)
23. particulate.mp. (84570)
24. nitrogen oxide.mp. (9147)
25. ozone.mp. (39435)
26. diesel.mp. (12621)
27. motor vehicles.mp. (6278)
28. 21 or 22 or 23 or 24 or 25 or 26 or 27 (493806)
29. 20 and 28 (328)
30. remove duplicates from 29 (221)

See Table 4.

**Table 4:** Correlations between sound levels and air pollution levels in urban areas (UK [2001–2003], Sweden [1992–1994] and [2004–2005], Denmark [1993–2006], Canada [1994–2002] and [2001], Switzerland [2002–2003], Spain [1995–2000] and [2008], Belgium [2009], Germany [2008], USA [2006–2007], Netherlands [1997–1998], [1987–1996] and [2006])

Studies	Characteristics	Noise		Air pollution		Correlation between noise and air pollution levels							
		Noise exposure (noise indicator)	Method	Air pollution exposure (air pollutants)	Method	NO_2_	NO_x_	PM_10_	PM_2.5_	UFP	Black smoke	O_3_	Black carbon
Dratva et al. (2012)	Cross-sectional *N*: 6,450 2002–2003 Switzerland	*L*_Day_ and *L*_night_	Dispersion model: SONABASE^M^	NO_2_ PM_10_	PolluMap Gaussian dispersion^M^	0.28–0.29		0.16–0.17					
Clark et al. (2012)	Cross-sectional *N*: 719 2001–2003 UK	*L*_Day_	Dispersion model: simplified form of the UK standard calculation of road traffic noise^M^	NO_2_	King’s college London Emissions Toolkit^M^	0.46							
Gan et al. (2012)	*N*: 445,868 1994–2002 Vancouver Canada	*L*_den_	Annual average of equivalent SPL for 24 h (CadnaA model using the EMME/2 for traffic volume)^**M**^	NO_2_ NO_X_ PM_2.5_	Annual average levels (land-use regression)	0.33	0.39	–	0.14	–	–	–	0.44
Sorensen et al. (2011)	*N*: 51,485 1995, 2000 and 2005 Copenhagen or Aarthus, Denmark	*L*_den_	Annual average of equivalent SPL for 24 h (sound plan model with the nordic prediction method, DANSIM and INM)^**M**^	NO_x_	Annual average levels (Danish AirGis modelling system)^**M**^	–	0.62	–	–	–	–	–	–
Foraster et al. (2011)	77 sites 2008 Girona, Spain	*L*_A,eq,24h_	Average of equivalent SPL for 24 h (Girona traffic noise model)^**M**^	NO_2_	Annual average estimated using NO_2_ monthly measurements^**M**^	0.62	–	–	–	–	–	–	–
Gan et al. (2010)	1 site 2009 Wolfstraat, Belgium	*L*_A,eq,15min_	15 min average of equivalent SPL	NO_2_ NO_X_ UFP	15 min average concentration (integrated multi gas measurement platform [Airpointer®, Recordum Austria])	0.29	0.46	–	–	0.38	–	–	–
Boogaard et al. (2009)	264 sites (132 routes) 2006 Netherlands	*L*_A,eq,1min_	1 min average SPL (measurement from 10:00 to 16:00)	PM_2.5_	One minute average concentration (condensation particle counter)	–	–	–	0.009	-	-	--	-
Weber (2009)	50 sites 2008 Essen, Germany	*L*_eq,20s_	Equivalent SPL for 20 s	PM_2.5_	Average of air pollution level at the beginning and the end of the noise monitoring period values (condensational particulate counter 1 s)	–	–	–	0.41–0.81	–	–	–	–
Selander et al. (2009)	*N*: 3,666 1970 to 1992–1994 Stockholm county, Sweden	*L*_A,eq,24h_	Annual average of equivalent SPL for 24 h (simplified version of the nordic prediction method)^**M**^	NO_2_	Annual average levels (Stockholm county dispersion model)^**M**^	0.6	–	–	–	–	–	–	–
Beelen et al. (2009)	*N*: 117,528 1986 home address with 2000–2001 noise imput data and 1987–1996 air pollutants input data Netherlands	*L*_den_	Annual average of equivalent SPL for 24 h (empara noise model)^**M**^	black smoke	Annual levels (land use and regression model (sum of regional, urban and local traffic contributions))^**M**^	–	–	–	–	–	0.24	–	–
Allen et al. (2009)	105 sites 2006–2007 Chicago and Riverside county USA	*L*_A,eq,5min_	Equivalent SPL for 5 min (measurement from 10:00 to 16:00)	NO_2_, NO_x_ and UFP	14 days average (Ogawa sampler)	0.16–0.62^a^	0.49–0.62^a^	–	–	0.22–0.41^a^	–	–	–
Davies et al. (2009)	103 sites 2001 Vancouver, Canada	*L*_A,eq,5min_	Equivalent SPL for 5 min (measurement at 103 sites from 8:00 to 18:00)	NO_2_ and NO_x_	14 days average (passive sampler)	0.53	0.64	–	–	–	–	–	–
de Kluizenaar et al. (2007)	*N*: 40,856 1997–1998 Sweden Groningen, Netherlands	*L*_den_	Annual average of equivalent SPL for 24 h (Urbis model [standard Karterings Method 2])^**M**^	PM_10_	Annual average levels (Netherlands’ standard Dutch models for local air pollution calculations: the street model CAR I and dispersion model “Pluim”)^**M**^	–	–	0.72	–	–	–	–	–
Persson et al. (2007)	*N*: 2,856 2004–2005 Scania, Sweden	*L*_A,eq,24h_	Annual average of equivalent SPL for 24 h (simplified version of the nordic prediction method)^**M**^	NO_X_	Modified Gaussian dispersion model (ENVIMAN)^M^		0.50						
ALNAP-(2006)^**1**^	*N*: NA NA Tyrol, Austria	*L*_den_	Annual average of equivalent SPL for 24 h (harmonoise model)^**M**^	NO_2_, and PM_10_	Annual average (network emission model [NEMO])^**M**^	0.12–0.48^a^	–	0.09–0.39^a^	–	–	–	–	–
Linares et al. (2006)	6 sites 1995–2000 Madrid, Spain	*L*_A,eq,24h_	Equivalent SPL for 24 h	NO_2_, SO_2_, O_3_, PM_10_ and NO_x_	Daily average (from the Madrid city air pollution network)	0.138	0.206	0.089	–	–	–	−0.275	–
Ising et al. (2004)	25 sites NA Germany	*L*_night_	Equivalent SPL from 22:00 to 6:00	NO_2_	Exposure from 58–93 h (palmes tube)	0.836	–	–	–	–	–	–	–
Tobias et al (2001)	6 sites 1995–1997 Madrid Spain	*L*_eq,24h_	Equivalent SPL for 24 h	NO_2_, SO_2_, O_3_ and NO_x_	Daily average (from the Madrid city air pollution network) except for O_3_ (1 h maximum value)	0.32	0.35	–	–	–	–	−0.42	–
Klæboe et al. (2000)	*N*: 1,028, 1,140, 1,097 1987, 1994 and 1996 Oslo, Norway	*L*_A,eq,24h_	Annual average of equivalent SPL for 24 h (nordic prediction method)^**M**^	NO_2_, PM_10_, PM_2.5_	3 month average of hourly estimations (dispersion model EPISODE)^**M**^	0.48	–	0.34	0.39	–	–	–	–
BEG-(1998)^b^	*N*: NA NA Tyrol, Austria	*L*_dn_	Annual average of equivalent SPL for 24 h (sound plan software)^**M**^	NO_2_ and PM_10_	NA (Gaussian propagation model) ^**M**^	0.63	–	0.61	–	–	–	–	–

^a^ Lowest and highest correlation of all the sampling sessions

^**M**^ Exposure asses using models

^b^ The description of those studies were available only in Lercher et al. 2011 (those studies are either not publish in English or French or are not publish at all)

*N* final sample size used for analysis

PM_2.5_ particles with a diameter of 2.5 μm or smaller, PM_10 _ particles with a diameter of larger than 2.5 μm but smaller than 10 μm, NO_2 _ nitrogen dioxide, NO_X _ nitrogen oxide, O_3_ Ozone, dB(A) a-weighted decibels, *L*_A,eq,24h_ integrated A-weighted sound level over 24 h, *L*_day_ integrated sound level over 16 h (0600–2200), *L*_night _ integrated A-weighted sound level over 8 h (2200–0600), *L*_A,eq,20s_ integrated A-weighted sound level over 20 s, *L*_A,eq,1min_ integrated A-weighted sound level over 1 min, *L*_A,eq,5min_ integrated A-weighted sound level over 5 min, *L*_A,eq,15min_ integrated A-weighted sound level over 15 min, *L*_dn_ integrated A-weighted sound level over 24 h (day, night) in which sound levels during the night (22h00–07h00) are increased by 10 dB(A), *L*_den_ integrated A-weighted sound level over 24 h (day, evening and night) in which sound levels during the evening (1900–2300 hours) are increased by 5 dB(A) and those during the night (2300–0700 hours) by 10 dB(A), Last validation 12th April 2012
